# Visible-light-sensitive titanium dioxide nanoplatform for tumor-responsive Fe2+ liberating and artemisinin delivery

**DOI:** 10.18632/oncotarget.17639

**Published:** 2017-05-05

**Authors:** Huijuan Zhang, Hongling Zhang, Xing Zhu, Xiaoge Zhang, Qianqian Chen, Jianjiao Chen, Lin Hou, Zhenzhong Zhang

**Affiliations:** ^1^ School of Pharmaceutical Sciences, Zhengzhou University, Henan Province, Zhengzhou, China; ^2^ Key Laboratory of Targeting Therapy and Diagnosis for Critical Diseases, Henan Province, Zhengzhou, China; ^3^ Collaborative Innovation Center of New Drug Research and Safety Evaluation, Henan Province, Zhengzhou, China

**Keywords:** Fe^2+^-dependent drug, visible-light-sensitive ROS production, tumor-responsive Fe^2+^ liberating

## Abstract

Artemisinin is a kind of Fe^2+^-dependent drugs. Artemisinin and Fe^2+^ co-transport systems can improve its anti-tumor effect. In this study, a visible light-sensitive nanoplatform (HA-TiO_2_-IONPs/ART) was developed. Detailed investigation demonstrated that HA-TiO_2_-IONPs/ART could realize Fe^2+^ and artemisinin synchronous co-delivery and tumor-responsive release. This feature enhanced the anti-tumor efficiency of artemisinin significantly. *In vitro* results proved that hyaluronic acid modification could improve the biocompatibility, dispersion stability and cytophagy ability of nanocarriers. Furthermore, this drug delivery system could generate reactive oxygen species under visual light irradiation. *In vitro* and *in vivo* experiments demonstrated that HA-TiO_2_-IONPs/ART combining with laser irradiation displayed the best anti-tumor efficacy. This study affords a promising idea to improve the curative efficiency of artemisinin analogs for cancer therapy.

## INTRODUCTION

Artemisinin (ART) is a sesquiterpene lactone antimalarial drug, which contains the specific endoperoxide bridge structure. This endoperoxide bridge can react with Fe^2+^ ions to generate radicals for killing parasite [1]. Nowadays, it has been reported that ART also has anti-tumor activity via the similar mechanism [2, 3]. In tumor cells, Fe^2+^ will react with endoperoxide bridge (-O-O-) to generate some strong alkylating agents, such as organic free radicals and electrophilic compounds. They can alkylate vital cellular components such as haem, glutathione, DNA, proteins or membranes to kill cancer cells [4]. To some extent, the anti-tumor efficacy of ART is positively correlated with Fe^2+^ amount in the targeted site. Though iron content in tumor is greater than normal cells, it is still much lower than that in erythrocytes [5]. So how to make these Fe^2+^-dependent drugs exert the greatest anti-tumor effect? Designing co-transport systems of Fe^2+^ ions and ART analogs will effectively solve this problem.

Another obstacle of ART for clinical application is its poor water solubility [6]. Nanovehicles can increase the solubility of hydrophobic drugs and offer proper particle size, so that they can accumulate in tumor tissues via EPR effect. Due to their excellent activity, high chemical stability, non-toxicity and low cost, titanium oxide (TiO_2_) nanoparticles have attracted much attention in drug delivery and cancer therapy fields recently. Especially, because TiO_2_ can create a high level of reactive radical oxygen species (ROS) under UV light irradiation, it has become a new class of photodynamic therapy (PDT) agent [7–9]. However, there are still some drawbacks hindering their wide application. They tend to absorb photons in UV region because of the wide band gap [10]. Their poor stability and high surface energy will cause coagulation under physiological conditions. It is difficult to achieve tumor-specific accumulation [9].

To address above problems, we introduced super-paramagnetic iron oxide nanoparticles (IONPs) to prepare magnetic titania nanocomposites (TiO_2_-IONPs). Firstly, TiO_2_ grafted with some transition metal oxide, such as Fe_2_O_3_ or Fe_3_O_4_, can serve as visible-light sensitive photocatalysts [7, 11]. Consequently, TiO_2_-IONPs can absorb visible light to generate ROS for tumor PDT. Secondly, it has been shown that iron oxide nanoparticles can be degraded in acid environments [12]. As we know, the tumor site is a slightly acidic environment [13]. Thus, Fe^2+^ ions will be easily released from TiO_2_-IONPs when ART-loaded nanovehicles reach the targeted tumor site. So it can deliver ART and Fe^2+^ ions into cancer cells synchronously, improving the anti-tumor efficacy.

**Figure 1:**
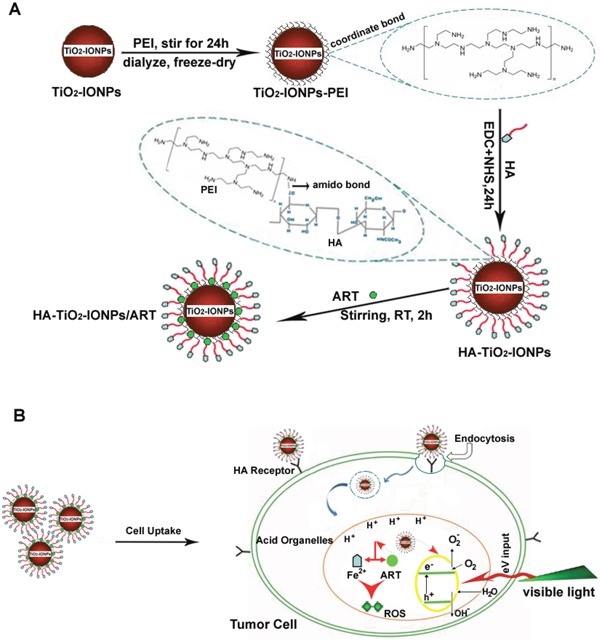
**(A)** Schematic illustration of the formation for this HA-TiO_2_-IONPs/ART delivery system; **(B)** The special multi-mechanism of HA-TiO_2_-IONPs/ART with visible light irradiation for tumor treatment.

Figure 1 showed the mechanism of this drug delivery system. As Figure 1A shown, polyethylenimine (PEI) was grafted on TiO_2_-IONPs by Fe-N coordination bond. Then hyaluronic acid (HA) was chemically bonded to the nanomaterials by amide linkages (HA-TiO_2_-IONPs), giving TiO_2_-IONPs good biocompatibility and active targeting ability. Finally, ART was loaded on this carrier to obtain a multi-functional drug delivery system (HA-TiO_2_-IONPs/ART). Figure 1B demonstrated the special anti-tumor mechanism of HA-TiO_2_-IONPs/ART under laser irradiation. When HA-TiO_2_-IONPs/ART incubated with tumor cells, they could enter cells by HA receptor-mediated endocytosis. Then they can reach those acid organelles, such as endosomes and lysosomes. Next, Fe^2+^ ions would release and act with the endoperoxide bridges of ART, to generate ROS for tumor killing effectively. At the same time, visible light (473-532 nm) was adopted to irradiate the tumor site. TiO_2_-IONPs nanovehicles would absorb this light energy, and then stimulate ROS generation for tumor PDT. This multifunctional drug delivery system was evaluated *in vitro* and *in vivo*.

## RESULTS

### Preparation of HA modified TiO_2_-IONPs nanoparticles

**Figure 2:**
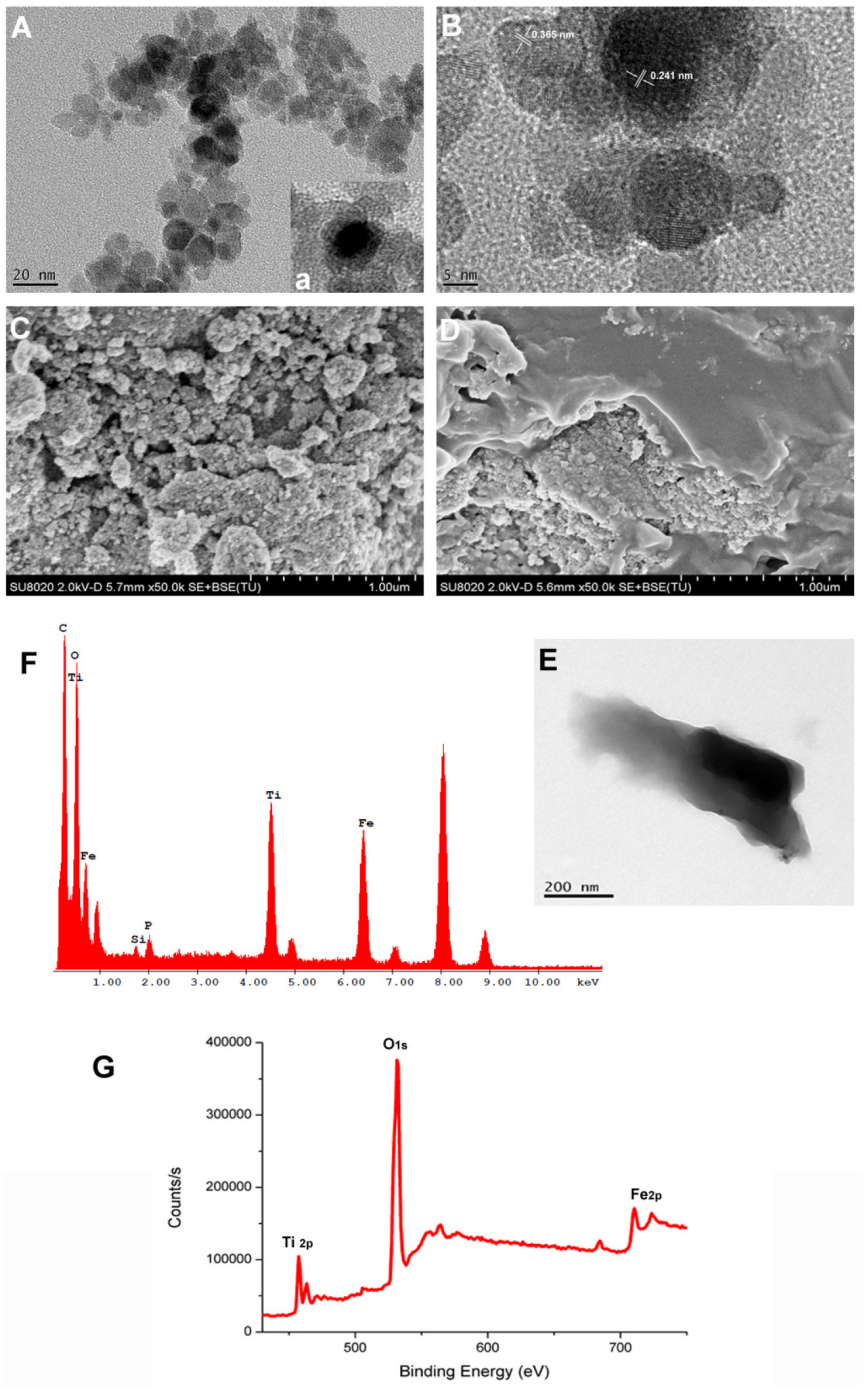
**(A)** TEM image of TiO_2_-IONPs; **(B)** High resolution TEM image of TiO_2_-IONPs; **(C)** SEM image of TiO_2_-IONPs; **(D)** SEM image of HA-TiO_2_-IONPs; **(E)** TEM image of HA-TiO_2_-IONPs; **(F)** Energy spectrum analysis of HA-TiO_2_-IONPs and **(G)** XPS test result of TiO_2_-IONPs.

The morphology and structure of TiO_2_-IONPs were characterized by SEM and TEM. As shown in Figure 2A-2C, TiO_2_-IONPs nanoparticles had an average diameter about 40 nm. The surface was rough because TiO_2_-IONPs were composed of many smaller particles. Because of the magnetic Fe_3_O_4_, some agglomerations of TiO_2_-IONPs appeared. It displayed a core-shell structure (Figure 2A (a)). The lattice fringes of high crystallographic TiO_2_ and Fe_3_O_4_ can be clearly identified (Figure 2B). The lattice constant of about 0.241 nm was corresponding to the (311) planes of Fe_3_O_4_ [14] and the lattice spacing of 0.365 nm was consistent with the (101) planes of anatase TiO_2_ (ICDD-JCPDS database, 21-1272) [15, 16]. Then XPS was performed to study the structural and chemical state of these elements. As Figure 2G shown, the doublet line of Fe corresponding to 2p_3/2_ and 2p_1/2_ were observed at 723.4 and 710.6 eV, respectively. The peaks at 457.6 and 463.4 eV were assigned to Ti (2p_3/2_) and Ti (2p_1/2_) core levels. This shoulder peak was assigned to formation of Ti–O–Fe bond in the interface of TiO_2_-IONPs, indicating the successful formation of TiO_2_-IONPs.

PEI dendrimer reacted with TiO_2_-IONPs by a strong complexing effect and endowed the vehicles amino groups. Then HA with carboxyl groups can be covalently linked to the nanocomposite via an amide bond. Figure 2D and 2E showed that there were obvious folds on TiO_2_-IONPs surface which accounted for the successful HA modification. Furthermore, element distribution of HA-TiO_2_-IONPs was examined by energy-dispersive X-ray spectroscopy (EDS) analysis. The result was shown in Figure 2F. Ti, Fe, O and C elements were found throughout the whole nanoparticle with high intensity. This indicated the successful formation of HA-TiO_2_-IONPs nanoparticles.

**Figure 3:**
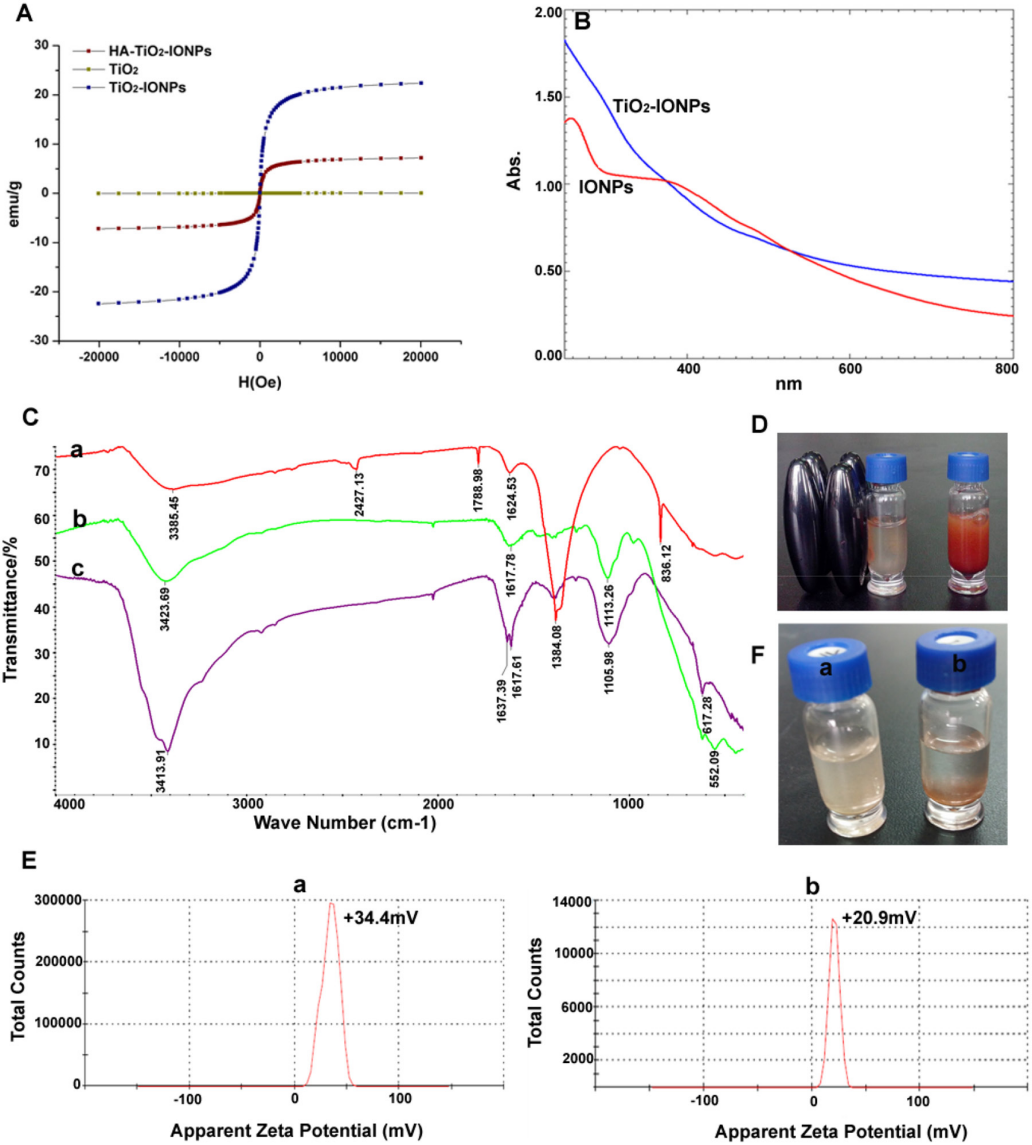
**(A)** Hysteresis curves of different nanoparticles; **(B)** Full spectrum scan; **(C)** Infrared spectrogram of a): TiO_2_-IONPs, b): PEI-TiO_2_-IONPs and c): HA-TiO_2_-IONPs; **(D)** The migration phenomenon under the magnetic field; **(E)** Apparent zeta potential of a): TiO_2_-IONPs and b): HA-TiO_2_-IONPs; **(F)** Water dispersibility of a): HA-TiO_2_-IONPs and b): TiO_2_-IONPs.

Figure 3B showed UV−vis absorption spectra of TiO_2_-IONPs aqueous dispersion. It was noted that TiO_2_-IONPs had absorption in the visible region. This property should make a positive contribution to photocatalytic activity under visible light irradiation. FT-IR analyses were carried out using KBr as reference to show the characteristic peaks (Figure 3C). For TiO_2_-IONPs hybrid, the profile showed a characteristic peak of Fe_3_O_4_ at 1384.08 cm^-1^ [17]. The typical peak at 500-1000 cm^-1^ related to Ti-O and Ti-O-Ti bonds. The peak at 3385.45 cm^-1^ was assigned to O-H stretching vibration [16, 18]. PEI grafting was confirmed by C-N (1113.26 cm^-1^) and N-H (1617.78 cm^-1^, 3423.69 cm^-1^) vibrations [19]. The chemical modification of HA achieved by forming amide linkage, which was confirmed by the typical C=O stretching vibration absorption at 1637.39 cm^-1^ and N-H bending vibration band at 1617.61 cm^-1^. The amide bonds were not easily cleavable, which assured its targeting ability *in vivo*.

The magnetic property was tested using a vibrating sample magnetometer at room temperature. As can been seen in Figure 3A, the saturation magnetization (Ms) value of TiO_2_-IONPs and HA-TiO_2_-IONPs were 22.38 and 7.19 emu/g. These nanoparticles could be easily drawn to the side wall of the vial by an external magnetic field (Figure 3D). This feature can be used to realize tumor magnetic targeting in practical applications. Obtained HA-TiO_2_-IONPs nanocarriers had good water dispersion (Figure 3F). The zeta potential of TiO_2_-IONPs and HA-TiO_2_-IONPs were 34.4 mV and 20.9 mV (Figure 3E), respectively.

### ART and Fe^2+^ released from the nanocarrier

#### ART loading

**Figure 4:**
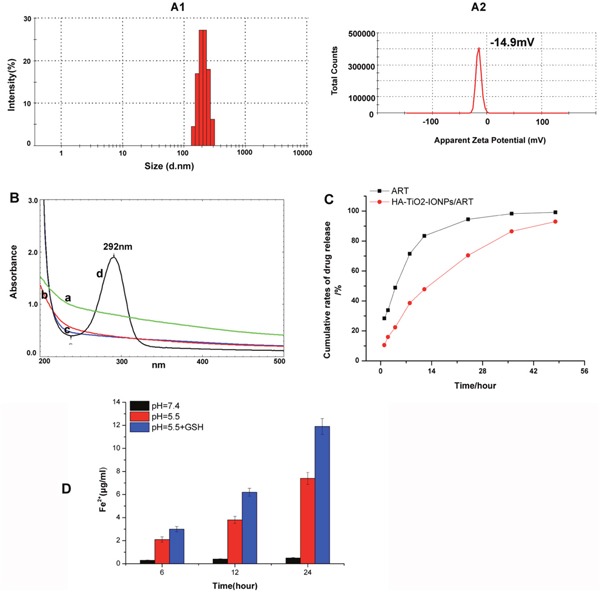
**(A)** Particle size distribution **(A1)** and apparent zeta potential **(A2)** of HA-TiO_2_-IONPs/ART; **(B)** UV-Vis spectrum of a): HA-TiO_2_-IONPs, b) HA-TiO_2_-IONPs/ART, c): HA-TiO_2_-IONPs after hydrolysis and d): HA-TiO_2_-IONPs/ART after hydrolysis; **(C)** Release curve of ART and HA-TiO_2_-IONPs/ART and **(D)** The amount of Fe^2+^ generated in different environments.

ART molecules were loaded onto HA-TiO_2_-IONPs by means of nanoprecipitation method. As Figure 4A shown, the particle size distribution and zeta potential of HA-TiO_2_-IONPs/ART were 205nm and -14.9mV, respectively. Those samples were assayed and confirmed by UV-Vis spectrophotometer before and after alkaline hydrolysis with NaOH (0.2%). The maximum absorption of ART was 205 nm. After being hydrolyzed by NaOH, its structure changed and the product had a significant absorption at 292 nm. So we chose this feature for ART loading conformation. As shown in Figure 4B, there were no additional peaks appeared for the carrier (a, c) before and after hydrolysis. While for HA-TiO_2_-IONPs/ART (b), a typical peak at 292 nm emerged after hydrolysis (d). The loading and encapsulation efficiency were calculated to be 27.5% and 76.0%, respectively.

#### ART and Fe^2+^ releasing

The antitumor activity of ART was related with Fe^2+^. So we explored ART and Fe^2+^ release characteristics. As seen in Figure 4C, ART could release from drug-loaded nanoparticles slowly and sustained. Next, we explored the capability of HA-TiO_2_-IONPs as Fe^2+^ donor. The result was demonstrated in Figure 4D. In tumor simulation environment (pH=5.5), the released Fe^2+^ increased along with time, reaching 7.4 μg/ml at 24h. However, only little Fe^2+^ (0.5 μg/ml) released within 24h incubation in neutral aqueous dispersion (pH=7.4). What's more, GSH also play a role in Fe^2+^-producing ability. As the result shown, GSH addition accelerated the release rate and amount of Fe^2+^. The released Fe^2+^ could even reach 11.9 μg/ml within the same time interval.

### *In vitro* anti-tumor efficacy

#### Cell uptake

**Figure 5:**
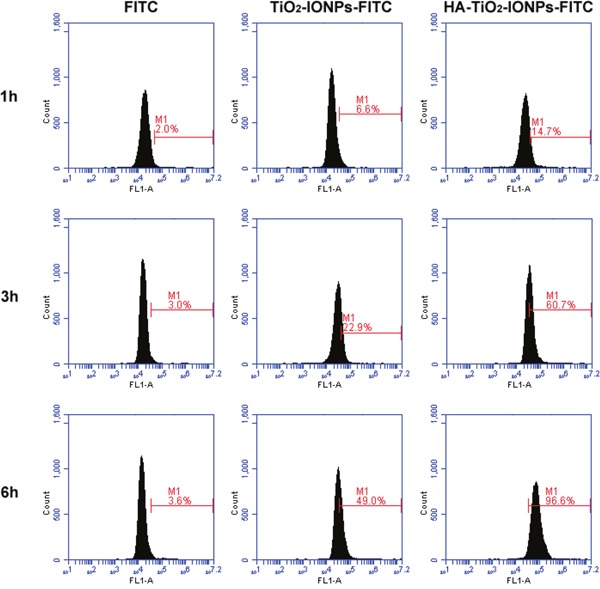
Cellular uptake of nanoparticles detected by flow cytometry

TiO_2_-IONPs and HA-TiO_2_-IONPs were labeled with FITC to investigate cellular uptake. After incubation for 1, 3 and 6 h, cells were collected for flow cytometry determination. The result was shown in Figure 5. The amount of endocytosed nanoparticles increased along with incubation time. Moreover, the amount of HA-TiO_2_-IONPs-FITC uptake by MCF-7 cells was statistically higher than that of TiO_2_-IONPs-FITC (P < 0.05). This was due to HA receptor mediated cellular endocytosis [4], leading to quicker and more nanocarrier's uptake by MCF-7 cells. For active targeting, a ligand is chosen to bind with a receptor overexpressed by tumor cells or tumor vasculature [20]. In this study, HA was grafted onto TiO_2_-IONPs. This is not only an available method to prevent magnetic nanoparticles aggregation but also beneficial to improve cytophagy ability.

#### Photocatalytic activity

**Figure 6:**
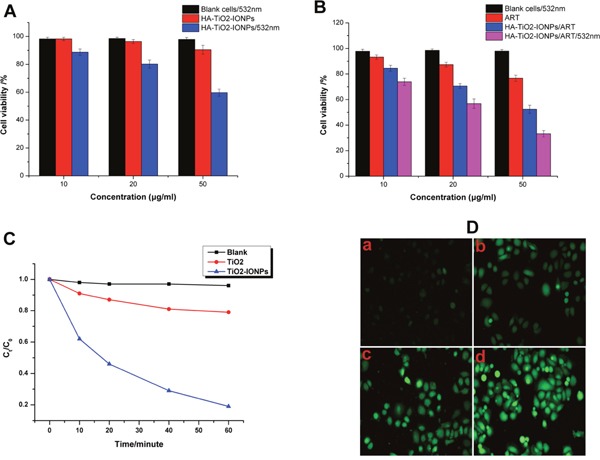
**(A)** Cell inhibition of HA-TiO_2_-IONPs *in vitro* with or without visible light irradiation; **(B)** Cell inhibition of ART and HA-TiO_2_-IONPs/ART with or without visible light irradiation; **(C)** Photocatalytic degradation of MB in the presence of TiO_2_-IONPs under visible light irradiation; **(D)** Intracellular ROS detection of a) control group, b) ART, c) HA-TiO_2_-IONPs/ART and d) HA-TiO_2_-IONPs/ART with visible light.

MB was chosen as a model organic pollutant to evaluate photocatalytic activity of TiO_2_-IONPs. As seen in Figure 6C, apparent degradation of MB was observed as soon as visible light irradiation (473-532 nm, 1.5 W/cm^2^). After irradiation for 60 min, approximate 79% MB was left in TiO_2_ control group, which was nearly 4.2-fold of that in TiO_2_-IONPs group. This comparative result indicated that compared with TiO_2_, the photocatalytic activity of TiO_2_-IONPs under visible light irradiation had been significantly enhanced.

#### ROS detection

ROS intracellular was detected using DCFH-DA fluorescent probe. The result was shown in Figure 6D. For ART group, there was weak green fluorescence observed in cancer cells. While in HA-TiO_2_-IONPs/ART group, significantly increased fluorescence intensity was observed. HA-TiO_2_-IONPs could provide Fe^2+^ in tumor microenvironment, which would interact with peroxide bridge of ART to produce ROS. This induced ROS increase in HA-TiO_2_-IONPs/ART group. What's more, when cells in HA-TiO_2_-IONPs/ART group were exposed to visible light, a much higher emission intensity of DCFH was recorded. This result was consistent with *Photocatalytic activity*, suggesting that HA-TiO_2_-IONPs could be acted as both drug vehicle and photosensitizer for tumor photodynamic therapy.

#### Cell survival and apoptosis test

To verify the biocompatibility and cytotoxicity of nanocarriers, MCF-7 cells were cultured with a series of different concentrations of HA-TiO_2_-IONPs for 24 h. As shown in Figure 6A, HA-TiO_2_-IONPs had no obvious cytotoxicity against cancer cells, with cell viability of 90.5% at 50μg/ml. For its PDT efficiency, a laser (473-532 nm, 1.5 W/cm^2^) was adopted. HA-TiO_2_-IONPs combining with laser presented a greatly enhanced cytotoxicity. The cell viability decreased to 59.7 %, indicating that HA-TiO_2_-IONPs could be used as a kind of inorganic photosensitizers for anti-tumor therapy.

Next, we investigated the cytotoxicity of ART and HA-TiO_2_-IONPs/ART (Figure 6B). A dose-dependent cytotoxicity was observed. Free ART had low anti-tumor efficiency on MCF-7 cells culturing for 24 h, even at a high concentration. Nevertheless, the cell viability dramatically decreased to around 50% when cells were incubated with HA-TiO_2_-IONPs/ART (50μg/ml). In order to investigate the enhanced PDT efficiency, after incubation with HA-TiO_2_-IONPs/ART, cancer cells were exposed to laser for 2 min. Remarkably enhanced anti-tumor effect was also observed. The cell viability declined significantly from 52.4% to 33.2%.

**Figure 7:**
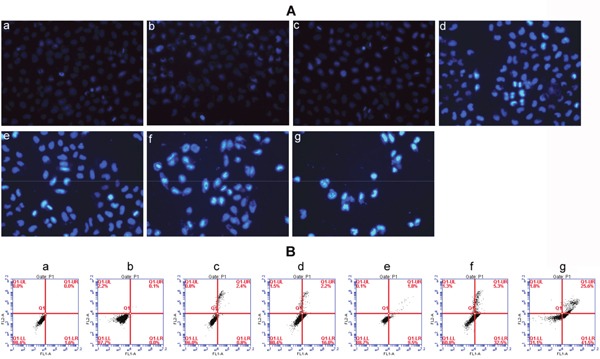
**(A)** Cell apoptosis detected by Hoechst 33342 staining of a) Blank cells, b) Blank cells/laser, c) HA-TiO_2_-IONPs, d) HA-TiO_2_-IONPs/laser, e) ART, f) HA-TiO_2_-IONPs/ART and g) HA-TiO_2_-IONPs/ART/laser; **(B)** Cell apoptosis detected by Flow cytometry of a) Blank cells, b) Blank cells/laser, c) HA-TiO_2_-IONPs, d) HA-TiO_2_-IONPs/laser, e) ART, f) HA-TiO_2_-IONPs/ART and g) HA-TiO_2_-IONPs/ART/laser.

Finally, Hoechst 33342 was used to detect cell apoptosis. Results were shown in Figure 7A. MCF-7 cells treated with HA-TiO_2_-IONPs (c) or laser only (b) both had negligible apoptosis. However, some cells showed chromatin condensation and bright blue signals in HA-TiO_2_-IONPs/laser group (d), suggested HA-TiO_2_-IONPs combining with visible light could induce MCF-7 cells apoptosis. Furthermore, in comparison to ART (e), HA-TiO_2_-IONPs/ART caused more chromatin condensation (f). When cells were exposed to visible light, approximately all cells showed chromatin condensation with shrunken and irregular shape. Some cells even showed chromatin fragmentation (g). Then flow cytometric analysis was carried out to detect apoptotic cell proportion (Figure 7B). There was no significant difference between blank cells (a) and blank cells /laser (b) groups. The amount of apoptotic cells in HA-TiO_2_-IONPs groups increased from 3.2% (c) to 18.2% (d) after laser irradiation. ART, HA-TiO_2_-IONPs/ART and HA-TiO_2_-IONPs/ART/laser groups showed t around 11.3% (e), 37.8% (f) and 67.1% (g) of apoptotic cells, respectively.

### *In vivo* tests

#### *In vivo* anti-tumor efficacy

**Figure 8:**
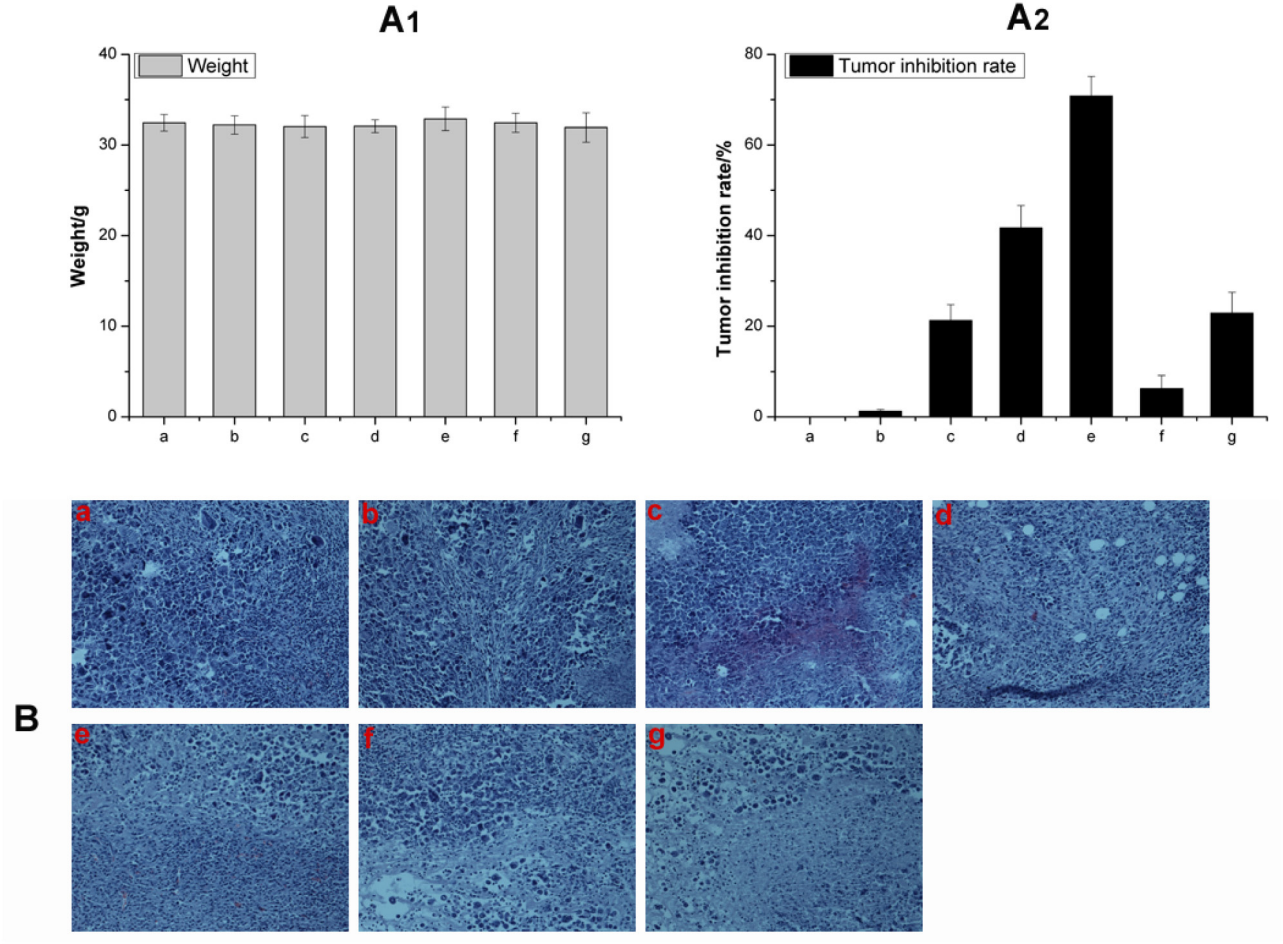
**(A)** The weight change **(A1)** and tumor inhibition rate **(A2)** of a) Control, b) Control/laser, c) ART, d) HA-TiO_2_-IONPs/ART, e) HA-TiO_2_-IONPs/ART/laser, f) HA-TiO_2_-IONPs and g) HA-TiO_2_-IONPs/laser; **(B)** H&E staining of tumor tissues of a) Control, b) Control/laser, c) HA-TiO_2_-IONPs, d) HA-TiO_2_-IONPs/laser, e) ART, f) HA-TiO_2_-IONPs/ART and g) HA-TiO_2_-IONPs/ART/laser.

Allowing for high toxicity usually leads to weight loss, the safety profiles of different formulations were evaluated by measuring the changes in body weight over time as shown in Figure 8A. The body weight of mice in saline, HA-TiO_2_-IONPs/ART and HA-TiO_2_-IONPs groups were 32.5, 32.0 and 32.4g at the end of the trial. There was no significant difference (P<0.05) among these three groups, implying that HA-TiO_2_-IONPs/ART would not cause significant systemic toxicity. Moreover, HA-TiO_2_-IONPs/ART group showed a tumor inhibition rate of 41.7%, while HA-TiO_2_-IONPs/ART combining visible light showed a tumor inhibition rate of 70%. ART, HA-TiO_2_-IONPs, HA-TiO_2_-IONPs/laser resulted in a tumor inhibition rate of 21.2%, 6.2% and 22.9%, respectively. The therapeutic efficacy was also evaluated by H&E staining. Seen from Figure 8B, cells in control and HA-TiO_2_-IONPs groups were in good condition. While in other groups, nucleus atrophy, necrosis and fragmentation were seen varying degrees. Symptoms including necrosis, karyotheca dissolving, and nucleolus disappearing was the most typical in HA-TiO_2_-IONPs/ART/laser group.

#### Biodistribution

**Figure 9:**
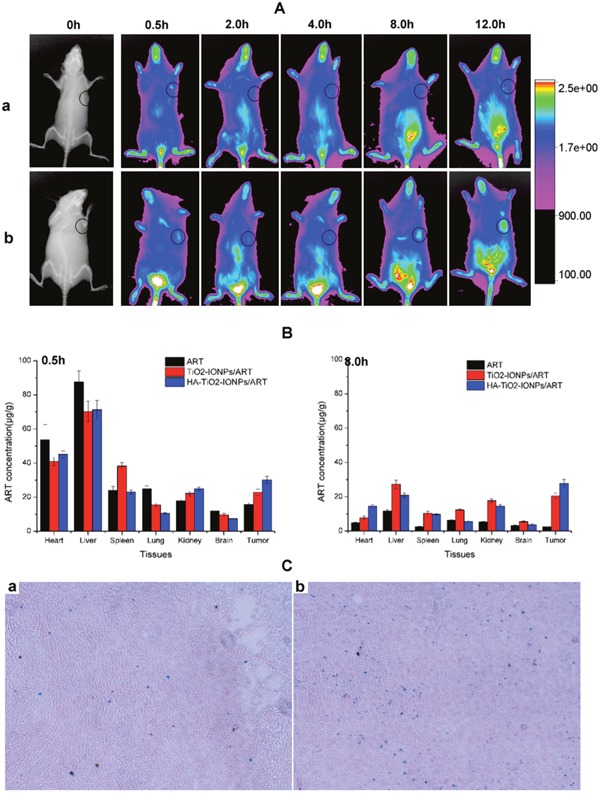
**(A)** *In vivo* NIR imaging of tumor-bearing mice intravenous injected with (a) free IR783 solution and (b) IR783-loaded HA-TiO_2_-IONPs at 0.5, 2, 4, 8 and 12h post injection; **(B)** Tissue distribution of ART, TiO_2_-IONPs/ART and HA-TiO_2_-IONPs/ART at 0.5h and 8h post injection; **(C)** The prussian blue staining images of (a) tumor tissue and (b) liver tissue for HA-TiO_2_-IONPs/ART group at 4h.

Figure 9A showed the real-time distribution of nanoparticles in tumor-bearing mice. The fluorescence signal in IR783control group was weak in whole-body and little distribution in tumor, revealing a bad targeting ability and a rapid clearance. Excitingly, IR783-loaded HA-TiO_2_-IONPs exhibited much stronger fluorescence intensity in tumor regions. As time increased, a preferential accumulation in tumor was observed within 12 h post-injection. In order to quantitatively evaluate HA-TiO_2_-IONPs/ART distribution *in vivo*, HPLC was used to determine ART concentration remaining in different organs. The results were summarized in Figure 9B. After administration, ART and its formulations could soon distribute in various tissues. 30min later, these three groups all exhibited a high ART concentration in tumor site. HA-TiO_2_-IONPs/ART formulations kept a high and effective drug level in tumor site up to 8 h. Moreover, prussian blue staining was carried out at 4h post-injection. Prussian blue can react with IONPs to produce blue compound. So the blue signals can be used to indicate the bio-distribution of these nanoparticles directly. As Figure 9C shown, HA-TiO_2_-IONP/ART could accumulate in the liver and tumor tissues. Because of their particle size (205nm), the nanoparticles would most likely accumulate in the liver. However, some nanoparticles may tend to aggregate in tumor tissues due to the EPR effect and active targeting ability. So the observed NIR signal in tumor may originate from two pathways. One source was HA-TiO_2_-IONP/IR783 accumulated in tumor. The other source was free IR783 dye, which was dissociated from the nanoparticles.

#### Pharmacokinetics experiment

**Figure 10:**
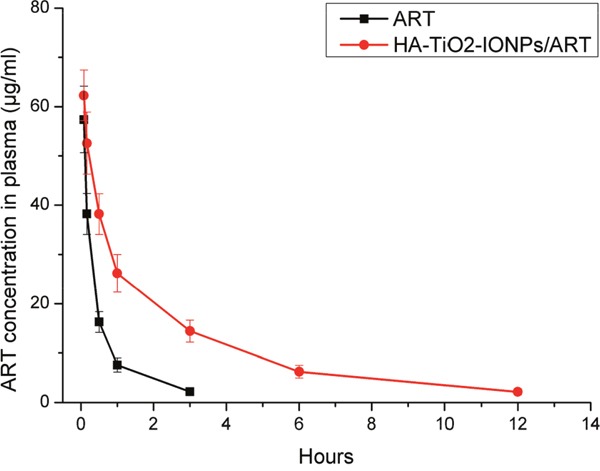
Plasma Concentration-time curve in mice of ART and HA-TiO2-IONPs/ART

We carried out pharmacokinetics experiment to study prolonged circulation of the nanoparticles, as shown in Figure 10 and Table 1. For ART group, drug concentration in plasma decreased faster than HA-TiO_2_-IONP/ART group. The drug concentration downward trend of HA-TiO_2_-IONP/ART was apparently slow. The area under curve (AUC) of HA-TiO_2_-IONP/ART (139.51μg/ml·h) was about 3.95-fold than that of ART (35.36μg/ml·h). The drug half-life (t_1/2_) of HA-TiO_2_-IONP/ART was about 2.85-fold than that of ART. The mean residence time (MRT) of HA-TiO_2_-IONP/ART was nearly four times longer than ART. All these data indicated that HA-TiO_2_-IONP/ART significantly increased blood circulation time of ART *In vivo*, supporting EPR effect.

**Table 1:** The main pharmacokinetic parameters of ART and HA-TiO_2_-IONPs/ART

Groups	AUC(μg/ml·h)	MRT(h)	T_1/2_(h)
ART	35.36	1.14	1.20
HA-TiO_2_-IONPs/ART	139.51	4.19	3.42

## DISCUSSION

Fe^2+^ within tumor can react with peroxide bridge structure (-O-O-) of ART to produce free radicals or electrophilic compounds for cancer therapy. So the anti-tumor efficacy of ART is positively correlated with Fe^2+^ amount in the targeted site. Herein, we synthesized HA-TiO_2_-IONPs/ART system and evaluated synchronous release characteristics of ART and Fe^2+^ in the simulate tumor environment. Hypoxia, acidity and high GSH concentration, are distinct and varied characteristics of solid tumors compared to normal tissue [21]. This special environment provided the co-transport possibility of ART and Fe^2+^. As seen in Figure 4, HA-TiO_2_-IONPs nanoparticles were not stable in acidic and reducing environment. Fe_3_O_4_ ingredient would be decomposed and reduced to Fe^2+^ ions slowly. These results suggested that HA-TiO_2_-IONPs/ART system can not only act as vehicles for ART but also as pH and GSH responsive Fe^2+^ reservoirs. Once HA-TiO_2_-IONPs/ART reached targeted tumor, Fe^2+^ can interact with ART to improve anti-cancer efficiency.

Futhermore, TiO_2_-IONPs can serve as visible-light sensitive photocatalysts [11]. The comparative result in Figure 6C showed that TiO_2_-IONPs nanocomposites had much higher degradation efficiency under visible light irradiation. The possible mechanism for this photocatalytic enhancement is as follows. IONPs is easily excited by visible light, generating photoelectrons and holes. Then these photoelectrons can be easily injected into the conduction band level of TiO_2_ owing to the potential difference, leaving holes in valence band of IONPs. As a result, charge carriers are separated successfully to improve the photocatalytic activity. Additionally, due to the small interface between IONPs and TiO_2_ in heterojunction structure, the recombination of light-induced electrons and holes will be decreased. This can enhance the quantum efficiency. Meanwhile, the generated conduction band electrons probably react with dissolved oxygen molecules to yield superoxide radical anions; the separated holes generate ·HO radicals [17]. Based on this mechanism, we explored TiO_2_-IONPs as photosensitizers for ROS generation and tumor therapy under visible light irradiation.

Polymer modification is an effective way to improve magnetic nanoparticles stability. HA is a naturally occurring polysaccharide, which exists widely in extracellular matrix [22]. Herein, HA was chemically grafted on TiO_2_-IONPs to make HA-TiO_2_-IONPs a biocompatible and non-immunogenic biomaterial (Figure 3D). Figure 4A showed this HA-TiO_2_-IONPs/ART formulation displayed good aqueous dispersion with a narrow particle size distribution around 205nm. The loading and encapsulation efficiency were calculated to be 27.5% and 76.0%, respectively. Although this encapsulation efficiency was lower than some nano-formulations reported [23], its small and uniform particle size was demonstrated to be advantageous for high accumulation in tumor tissue by EPR effect [24], which made it suit for the practical applications.

Next, *in vitro* and *in vivo* experiments were carried out to evaluate the anti-tumor effect. As a tumor targeting ligand, HA could improve tumor targeting ability and enhance cellular endocytosis. *In vivo* results showed HA-TiO_2_-IONPs/ART kept a high and effective drug level in tumor site and extended blood circulation time. Figure 6B and Figure 8 showed the anti-tumor effect *in vitro* and *in vivo*, respectively. ART had low anti-tumor efficiency even at a high concentration, while HA-TiO_2_-IONPs/ART group showed a significantly enhanced anti-tumor effect. After combing with laser irradiation, the tumor suppression effect was further improved. This demonstrated phototherapy application of HA-TiO_2_-IONPs *in vivo* was successful. When HA-TiO_2_-IONPs/ART reached targeted site, the tumor-sensitive carrier can generate and release Fe^2+^. Then ART and Fe^2+^ could react in the same loci for ROS generation. So mice treated with HA-TiO_2_-IONPs/ART showed better tumor suppression effect than ART group. These results indicated that the Fe^2+^ and ART co-delivery system combining with PDT could get the best anti-tumor effect.

## MATERIALS AND METHODS

### Materials

Tetrabutyl orthotitanate (C_16_H_36_O_4_Ti, ≥ 98%) was obtained from Tokyo Chemical Industry Co. Ltd (Tokyo, Japan). Artemisinin (ART, > 99.0%) was purchased from Create-Life Biotech Limited Company (Zhengzhou, China). Ferrous sulfate tetrahydrate (FeSO_4·_4H_2_O, > 99.0%), ferric chloride hexahydrate (FeCl_3·_6H_2_O, > 99.0%), oleic acid (C_18_H_34_O_2_, > 98.0%) and absolute ethanol ( > 99.5%) were bought from Sinopharm Chemical Reagent Co. Ltd (Shanghai, China). Sodium hyaluronate (HA, MW≈12000, > 98%) was bought from Bloomage Freda Biopharm Co. Ltd (Jinan, Shandong). Polyethyleneimine (PEI, MW≈700, > 98.0%), N-(3-dimethylamino propyl -N′-ethylcarbodiimide) hydrochloride (EDC_·_HCl), N-Hydroxysuccinimide (NHS), fluorescein isothiocyanate (FITC), sulforhodamine B (SRB), dimethyl sulfoxide (DMSO), triethylamine were obtained from Sigma-Aldrich (St Louis, MO, USA). Penicillin, streptomycin and fetal bovine serum were bought from Life Technologies (Carlsbad, CA, USA).

### Preparation of HA modified TiO_2_-IONPs nanoparticles

#### Synthesis of TiO_2_-IONPs

In total, FeCl_3_.6H_2_O (5.4 g) and FeSO_4_·7H_2_O (2.9 g) were added to a beaker containing 350 ml of ultrapure water to get an aqueous solution. Then adjust pH to 2 with hydrochloric acid (HCl). After that, 0.43 g of trisodium citrate was added into this reaction mixture. Stir this solution evenly and adjust pH to 9 by adding ammonia drop by drop at 70-80 °C. Discard the supernatant after standing for 30 min. Wash the precipitate with distilled water for several times, followed by ethanol until neutral. Dissolve 5 ml of tetrabutyl titanate in 15 ml of anhydrous ethanol. Stir constantly until a sol formed. Then drop into the aforesaid magnetic liquid slowly. Adjust pH to 4~5 with 1 M nitric acid at 100-110 °C, and continue stir until the sol changed into a gel. Dry the gel at 80°C for 2 h and finely grind in an agate mortar. The products were calcined at 450°C for 2 h in a muffle furnace.

#### Synthesis of HA-TiO_2_-IONPs

Add TiO_2_-IONPs (150 mg) into PEI aqueous solution (1.5 mg/ml) and stir for 24 h at room temperature. After dialyzed, the solution was lyophilized to obtain TiO_2_-IONPs-PEI powder. Disperse this powder in formamide (50 ml). After that, add HA (90 mg), EDC_·_HCl (173 mg) and NHS (103 mg) into this solution. Stir for 24 h at room temperature. Cool the reaction solution with excess pre-cooled acetone (3~4 times the amount of reaction solution) and centrifuge at 10, 000 rpm for 15 min. Finally the precipitation was re-dissolved with water and dialyzed (MW = 12, 000) to remove EDC·HCl, NHS and free HA. Finally, the synthesis products (HA-TiO_2_-IONPs) were freeze-dried in vacuum for 48 h.

#### Characterization

DLS (Zetasizer Nano ZS-90, Malvern, UK), SEM (Quonxe-2000, FEI, Netherlands) and TEM (Tecnai G2 20, FEI) were used for characterizing particle size, zeta potential and morphological of HA-TiO_2_-IONPs, respectively. The optical properties of HA-TiO_2_-IONPs were characterized using a ultra-violet visible (UV-vis) spectrometer (Lambda35, Perkin-Elmer, USA) and a Nicolet iS10 spectrometer (FI-TR, Thermo).

### ART and Fe^2+^ released from the nanocarrier

#### Drug loading and releasing

To prepare ART-loadedmagnetic nanoparticles, HA-TiO_2_-IONPs carriers (6 mg) were dispersed in ultrapure water (12 ml) by ultrasonic techniques. Then add predetermined amount of ART (6.0 mg), which dissolved in 1.0 ml ethanol, to the above dispersion and stir for 24 h at room temperature. After that, the suspension was dialyzed for 8 h (MW = 3,500) in dialysate (distilled water: ethanol = 9:1) to remove unloaded drug. The obtained preparation (HA-TiO_2_-IONPs/ART) was lyophilized to store. To measure loading efficacy, dilute the nanosuspension with 10 times the volume of ethanol and sonicate to ensure ART dissolving completely. Then centrifuge to separate carrier and drug. Thereafter, hydrolyze ART with 4-fold volume of NaOH (0.2%) for 30 min at 50±1 °C. Measure the absorbance at 292 nm to calculate drug loading efficacy by the standard method. The drug release test *in vitro* was performed in PBS containing 10% ethanol at 37.0±0.5 °C. In brief, HA-TiO_2_-IONPs/ART dispersion liquid was transferred into a dialysis bag (MW cutoff = 3,500). Then put the dialysis bag into 50 ml of medium with a gently stirring rate (100 rpm). At predetermined time points, 0.2 ml of dialyzate was drawn for quantification analysis and replaced by the same volume of fresh medium to maintain sink conditions. The released ART was determined after hydrolysis.

#### Fe^2+^ generated in different environment

Iron Assay Kit (Sigma-Aldrich, MO, USA) was used to determine Fe^2+^ concentration. Dissolve HA-TiO_2_-IONPs in water with different acidity (pH = 5.5 or 7.4) and GSH content to obtain solutions with the same carrier concentration (100 μg/ml). These samples were labeled as ① (pH = 5.5 without GSH), ② (pH = 7.4 without GSH) and ③ (pH = 5.5 with 5 mM GSH). Shake them on a horizontal shaker (100 rpm) at room temperature. At 4 and 8h, 50 μl sample was drawn and transferred to 96-well plates. Bring samples to a final volume of 100 μl with iron assay buffer. To measure ferrous iron, add 5 μl of iron assay buffer to each well. Mix well and incubate the reaction for 30 min at room temperature in the dark. Add 100 μl of iron probe to each well containing test samples. Mix well and incubate for 60 min without light. At last, measure the absorbance at 593 nm.

### *In vitro* efficacy

#### Photocatalytic activity

The Photocatalytic activity of TiO_2_-IONPs was evaluated by photodegradation of methyl blue (MB, 50 mg/L) in aqueous solution under visible light irradiation.Catalytic reactions were conducted in a 100 ml flask with constant mechanical agitation at room temperature, in the presence ofTiO_2_-IONPs (100 mg in 50 ml of solution). The suspension composed of MB and TiO_2_-IONPs was stirred in the dark for 30 min to achieve absorption-desorption equilibrium. Then dispersions were illuminated using a laser device (473-532 nm) with an intensity of 1.5 W/cm^2^. Aliquots (3.0 ml) were taken and separated from the photocatalysts by filtration through a 0.22 μm polyvinylidene fluoride (PVDF) syringe filter every ten minute. The filtrate was used to determine MB concentration by a UV-VIS spectrometer at 646 nm, to get a time-dependent change of undegraded MB.

#### Detection of intracellular ROS

The ROS generation ability of HA-TiO_2_-IONPs was assessed using DCFH-DA Assay Kit. MCF-7 cells were seeded in 6-well culture plates. The preparations (ART or HA-TiO_2_-IONPs/ART) were added to each well with the same concentration (ART: 20 μg/ml; HA-TiO_2_-IONPs: 40 μg/ml) and incubated for 12 h. Then culture medium containing drugs was removed and DCFH-DA was loaded into the cells for 30 min. Thereafter, rinse with *PBS* for three times. For laser irradiation group, cells were exposed to laser for 2 min with density of 1.5 W/cm^2^. Finally, fluorescence images were acquired using a Fluorescence Microscope.

#### Proliferation assay

Briefly, 200 μl of MCF-7 cells suspension (5×10^3^ cells/well) was added into a 96-well plate and allowed cells to attach. After that, discard the medium and load different concentrations of ART, HA-TiO_2_-IONPs or HA-TiO_2_-IONPs/ART. The proliferation of cells was evaluated after 24 h. In order to evaluate anti-proliferative effects of HA-TiO_2_-IONPs and HA-TiO_2_-IONPs/ART in the presence of visible light, a laser (473-532 nm, 1.5 W/cm^2^) was used to irradiate the 96-well plate for 2 min. At the end of the treatment, cytotoxicity was analyzed by Sulforhodamine B (SRB) assay.

#### Cellular uptake

HA-TiO_2_-IONPs were labled with FITC to explore the cellular uptake ability. The internalization of FITC-labeled HA-TiO_2_-IONPs was evaluated by flow cytometry. MCF-7 cells were treated with 10 μg/ml FITC-labeled TiO_2_-IONPs or FITC-labeled HA-TiO_2_-IONPs for 1 h, 3 h and 6 h. Subsequently, treated cells were washed with PBS to remove those nanocarriers not uptaken by cancer cells. Finally, cells were harvested by trypsinization and detected with flow cytometry.

#### Cell apoptosis

Hoechst 33342 staining was used to detect cell apoptosis. Briefly, 3 ml of MCF-7 cells suspension (5×10^4^ cells/well) was added into a 6-well plate and allowed cells to attach for 24 h. After that, discard the medium and load different formulations of ART, HA-TiO_2_-IONPs or HA-TiO_2_-IONPs/ART. Incubate cells for a further 24 h. Finally, cells were washed with PBS and stained with Hoechst 33342. The results were acquired and recorded by a Fluorescence Microscope. For laser groups, MCF-7 cells in the 6-well plate were under laser exposure (473-532 nm, 1.5 W/cm^2^) for 2 min. Furthermore, flow cytometric analysis was carried out to detect apoptotic cell proportion by using an Annexin-V-Fluos Staining kit. After treated, the cells were collected and stained with 5μl recombinant human anti-Annexin-V-FITC and 5μl of propidium iodide (PI). After reaction at room temperature in the dark for 10 min, all the samples were immediately detected by using flow cytometry (FCM, Epics XL, COULTER, USA).

### *In vivo* tests

#### Xenograft tumor mouse model

All animal experiments were performed under a protocol approved by Henan laboratory animal center. The S180 tumor models were generated by subcutaneous injection of 2×10^6^ cells into the right shoulder of female BALB/c mice (18-20 g, Henan laboratory animal center). The mice were used for *in vivo* anti-tumor experiment while tumor volume reached ~100 mm^3^ (about 7 days after tumor inoculation).

#### *In vivo* anti-tumor efficacy

The tumor-bearing mice were divided into 6 groups (six mice per group to minimize the differences of weights and tumor sizes in each group). The mice were administered with (1) saline, (2) HA-TiO_2_-IONPs, (3) ART, (4) HA-TiO_2_-IONPs/ART, (5) HA-TiO_2_-IONPs/Laser and (6) HA-TiO_2_-IONPs/ART/laser by tail vein injection every 2 days for 10 days, respectively (ART dose: 50 mg/kg). Tumor regions in groups (5) and (6) were irradiated with laser (473-532 nm, 1.5W/cm^2^) for 2 min at 4 h post-injection. At the end of experiment, animals were weighted and sacrificed. Tumor tissues were taken out and weighed. Tumor inhibition rate was calculated by formulation: (W_control_-W_test_)/ W_control_×100%. W_control_ and W_test_ represented tumor weights in saline and medication administration groups. Then, tumor tissues of each group were soaked in 10% formalin solution, embedded with paraffin for hematoxylin and eosin (H&E) staining. Morphological changes were observed under microscope (Eclipse 80i, Nikon, Japan).

#### Biodistribution studies

Tumor-bearing mice were treated by tail vein injection with ART solution, TiO_2_-IONPs/ART or HA-TiO_2_-IONPs/ART at a matched dose of 50 mg/kg. At times points, six animals were killed and tissues (heart, liver, spleen, lung, kidney, brain and tumor) were homogenized in saline with W/V 1: 3. Then samples were extracted by diethyl ether. After dried, the residue was redissolved with methyl alcohol. Finally, ART was determined by HPLC at 210 nm, with mobile phase of acetonitrile/water: 52/48. What’ more, tissues were stained with prussian blue (Prussian blue staining kits, Solarbio) to indicate the bio-distribution of HA-TiO_2_-IONPs directly.

#### NIR imaging

Noninvasive NIR imaging was used to visually monitor biodistribution and accumulation in tumor of this delivery system. HA-TiO_2_-IONPs were labeled with IR783, a kind of NIRF dye. The model mice were intravenously injected with IR783 solution and IR783-loaded HA-TiO_2_-IONPs, with the same dosage of IR783 (1.6 mg/kg). After injection for 0.5, 2, 4, 8, and 12 h, a Kodak *in vivo* imaging system FX PRO (Kodak, USA) with an excitation bandpass filter at 700 nm and an emission at 830 nm was used to record and analysis the results.

#### Pharmacokinetics experiment

0.8 ml blood was drawn from eyes of healthy BALB/c mice after treatment with ART and HA-TiO_2_-IONPs/ART (50 mg/kg) for 0.08ˋ 0.17ˋ 0.5ˋ 1ˋ 3ˋ 6 and 12 h. Centrifuge and the supernatant (0.2 ml) were placed into centrifuge tubes. Then ART in samples were extracted by diethyl ether. After dried, the residue was redissolved with methyl alcohol. Finally, ART was determined by HPLC at 210 nm, with mobile phase of acetonitrile/water: 52/48.

## CONCLUSION

In summary, we prepared a visible light-sensitive and tumor-responsive Fe^2+^ and ART co-delivery system. HA was chemically modified on TiO_2_-IONPs to improve its biocompatibility and dispersion stability. Furthermore, results *in vitro* proved that HA can improve the cytophagy ability as a tumor targeting ligand. *In vivo* results showed HA-TiO_2_-IONPs/ART kept a high and effective drug level in tumor site and extended blood circulation time. HA-TiO_2_-IONPs/ART system can not only act as vehicles for ART but also as pH and GSH responsive Fe^2+^ reservoirs. Once HA-TiO_2_-IONPs/ART reached targeted tumor, Fe^2+^ can interact with ART to improve anti-cancer efficiency. Combining with visual light irradiation, the system displayed the best tumor inhibitory efficacy *in vitro* and *in vivo*.Although further study should be deeply investigated to reveal the mechanism, HA-TiO_2_-IONPs/ART system presented itself as a promisingcandidate for tumor therapy.

## References

[1] Ho WE, Peh HY, Chan TK, Wong WS (2014). Artemisinins: pharmacological actions beyond anti-malarial. Pharmacology & therapeutics.

[2] Shahbazfar AA, Zare P, Ranjbaran M, Tayefi-Nasrabadi H, Fakhri O, Farshi Y, Shadi S, Khoshkerdar A (2014). A survey on anticancer effects of artemisinin, iron, miconazole, and butyric acid on 5637 (bladder cancer) and 4T1 (Breast cancer) cell lines. Journal of cancer research and therapeutics.

[3] Tilaoui M, Mouse HA, Jaafari A, Zyad A (2014). Differential effect of artemisinin against cancer cell lines. Natural products and bioprospecting.

[4] Zhang H, Hou L, Jiao X, Ji Y, Zhu X, Zhang Z (2015). Transferrin-mediated fullerenes nanoparticles as Fe(2+)-dependent drug vehicles for synergistic anti-tumor efficacy. Biomaterials.

[5] Shterman N, Kupfer B, Moroz C (1991). Comparison of transferrin receptors, iron content and isoferritin profile in normal and malignant human breast cell lines. Pathobiology.

[6] Chen YF, Lin XF, Park H, Greever R (2009). Study of artemisinin nanocapsules as anticancer drug delivery systems. Nanomedicine.

[7] Liu M, Inde R, Nishikawa M, Qiu XQ, Atarashi D, Sakai E, Nosaka Y, Hashimoto K, Miyauchi M (2014). Enhanced Photoactivity with Nanocluster-Grafted Titanium Dioxide Photocatalysts. Acs Nano.

[8] Nakamura M, Ono A, Bae E, Murakami N, Ohno T (2013). Improvement of visible light responsivity of rutile TiO2 nanorods by site-selective modification of iron(III) ion on newly exposed faces formed by chemical etching treatment. Appl Catal B-Environ.

[9] Zhang H, Shi RH, Xie AJ, Li JC, Chen L, Chen P, Li SK, Huang FZ, Shen YH (2013). Novel TiO2/PEGDA Hybrid Hydrogel Prepared in Situ on Tumor Cells for Effective Photodynamic Therapy. Acs Appl Mater Inter.

[10] Yin M, Ju E, Chen Z, Li Z, Ren J, Qu X (2014). Upconverting nanoparticles with a mesoporous TiO2 shell for near-infrared-triggered drug delivery and synergistic targeted cancer therapy. Chemistry.

[11] Mahadik MA, Shinde SS, Mohite VS, Kumbhar SS, Moholkar AV, Rajpure KY, Ganesan V, Nayak J, Barman SR, Bhosale CH (2014). Visible light catalysis of rhodamine B using nanostructured Fe2O3, TiO2 and TiO2/Fe2O3 thin films. J Photoch Photobio B.

[12] Arbab AS, Wilson LB, Ashari P, Jordan EK, Lewis BK, Frank JA (2005). A model of lysosomal metabolism of dextran coated superparamagnetic iron oxide (SPIO) nanoparticles: implications for cellular magnetic resonance imaging. Nmr Biomed.

[13] Ulbrich K, Subr V (2004). Polymeric anticancer drugs with pH-controlled activation. Adv Drug Deliver Rev.

[14] Zhang SZ, He W, Zhang XD, Yang GH, Ma JY, Yang XN, Song X (2015). Fabricating Fe3O4/Fe/Biocarbon Fibers using Cellulose Nanocrystals for High-Rate Li-ion Battery Anode. Electrochim Acta.

[15] Xu JW, Gao ZD, Han K, Liu YM, Song YY (2014). Synthesis of Magnetically Separable Ag3PO4/TiO2/Fe3O4 Heterostructure with Enhanced Photocatalytic Performance under Visible Light for Photoinactivation of Bacteria. Acs Appl Mater Inter.

[16] Aguilar CAH, Pandiyan T, Arenas-Alatorre JA, Singh N (2015). Oxidation of phenols by TiO2-Fe3O4-M (M = Ag or Au) hybrid composites under visible light. Sep Purif Technol.

[17] Yang XL, Chen W, Huang JF, Zhou Y, Zhu YH, Li CZ (2015). Rapid degradation of methylene blue in a novel heterogeneous Fe3O4@rGO@TiO2-catalyzed photo-Fenton system. Sci Rep.

[18] Guo MY, Liu FZ, Leung YH, Ng AMC, Djurisic AB, Chan WK (2013). TiO2-carbon nanotube composites for visible photocatalysts - Influence of TiO2 crystal structure. Curr Appl Phys.

[19] Shi JJ, Zhang HL, Wang L, Li LL, Wang HH, Wang ZZ, Li Z, Chen CQ, Hou L, Zhang CF, Zhang ZZ (2013). PEI-derivatized fullerene drug delivery using folate as a homing device targeting to tumor. Biomaterials.

[20] Li JC, He Y, Sun WJ, Luo Y, Cai HD, Pan YQ, Shen MW, Xia JD, Shi XY (2014). Hyaluronic acid-modified hydrothermally synthesized iron oxide nanoparticles for targeted tumor MR imaging. Biomaterials.

[21] Chen YF, Lin XF, Park H, Greever R (2009). Study of artemisinin nanocapsules as anticancer drug delivery systems. Nanomedicine.

[22] Yoon HY, Koo H, Choi KY, Lee SJ, Kim K, Kwon IC, Leary JF, Park K, Yuk SH, Park JH, Choi K (2012). Tumor-targeting hyaluronic acid nanoparticles for photodynamic imaging and therapy. Biomaterials.

[23] Chen D, Bobko AA, Gross AC, Evans R, Marsh CB, Khramtsov VV, Eubank TD, Friedman A (2014). Involvement of Tumor Macrophage HIFs in Chemotherapy Effectiveness: Mathematical Modeling of Oxygen, pH, and Glutathione. Plos One.

[24] Danhier F, Feron O, Preat V (2010). To exploit the tumor microenvironment: Passive and active tumor targeting of nanocarriers for anti-cancer drug delivery. J Control Release.

